# A novel geometry image to accurately represent a surface by preserving mesh topology

**DOI:** 10.1038/s41598-021-01722-4

**Published:** 2021-11-19

**Authors:** Sheng Zeng, Guohua Geng, Hongjuan Gao, Mingquan Zhou

**Affiliations:** Northwest university, Xi’an, China

**Keywords:** Mathematics and computing, Computational science, Computer science

## Abstract

Geometry images parameterise a mesh with a square domain and store the information in a single chart. A one-to-one correspondence between the 2D plane and the 3D model is convenient for processing 3D models. However, the parameterised vertices are not all located at the intersection of the gridlines the existing geometry images. Thus, errors are unavoidable when a 3D mesh is reconstructed from the chart. In this paper, we propose parameterise surface onto a novel geometry image that preserves the constraint of topological neighbourhood information at integer coordinate points on a 2D grid and ensures that the shape of the reconstructed 3D mesh does not change from supplemented image data. We find a collection of edges that opens the mesh into simply connected surface with a single boundary. The point distribution with approximate blue noise spectral characteristics is computed by capacity-constrained delaunay triangulation without retriangulation. We move the vertices to the constrained mesh intersection, adjust the degenerate triangles on a regular grid, and fill the blank part by performing a local affine transformation between each triangle in the mesh and image. Unlike other geometry images, the proposed method results in no error in the reconstructed surface model when floating-point data are stored in the image. High reconstruction accuracy is achieved when the xyz positions are in a 16-bit data format in each image channel because only rounding errors exist in the topology-preserving geometry images, there are no sampling errors. This method performs one-to-one mapping between the 3D surface mesh and the points in the 2D image, while foldovers do not appear in the 2D triangular mesh, maintaining the topological structure. This also shows the potential of using a 2D image processing algorithm to process 3D models.

## Introduction

The geometry images (GIMs) represent the surfaces using the completely regular structure^1^. Resampling geometry onto an image helps to apply signal-processing operations and in rendering on the GPU to maximise throughput^2,3^. The index of vertex coordinates is implicit in a 2D array, which offers a number of benefits, such as encoding geometry images using a traditional image compression algorithm. It combines the processing methods for both the geometry and the image.

The classical geometry image method cuts an arbitrary surface along an appropriate set of paths, creating a mesh with a single boundary (a topological disk) and parameterising the mesh onto the square domain of the geometry image. By choosing extreme vertices and identifying triangles with the maximum geometric stretch, the shortest path to the new rectangular boundary is determined. Then, a uniform 2D sampling grid is overlaid, and the samples are projected back onto the surface. The GIM improves the reconstruction accuracy by increasing the sampling resolution. The GIM stores the xyz positions in a 12-bit data format per channel, and the help of the normal is required for geometry reconstruction. Two factors generate errors, i.e., the sample point is not coincident with integer grid points; thus, the xyz positions are not inaccurate, and the round-off errors are over the coordinates of vertices. There are errors in storing 3D data with images, which means that the original shape will be changed when reconstructing 3D mesh from the three-channel data of image data and topological relationship , that is, the image can not accurately represent the 3D model.

Many geometry images focus on the mesh parameterisation or resampling process to reduce the reconstruction errors and preserve the geometric details. The basic idea of the improved method is to map the mesh piecewise onto several charts, record vertices to appear repeatedly in an image array, or optimise the sequences of triangles. All of the images need to have a resampling step to ensure that the data stored in the image can be as close as possible to the original 3D data. However, regardless of how the sampling resolution is increased, it is impossible to align all integer grid points and parametric points. resampling inevitably changes the connectivity of vertices, it limits the introduction of traditional image processing algorithms.

The one-to-one mapping between the 2D plane and the surface embedded in the 3D space enables processing of the 3D model as easily as when the surface is flat. To ensure the bijective correspondence between the input 3D surfaces and the geometry images, we propose topology-preserving geometry images (TPGIMs). This parameterisation method forcefully moves the vertices to an integer position and does not generate repetition vertices or fold triangular faces. Therefore, the floating-point data stored in the integer point position are entirely consistent with the 3D mesh. The 16-bit data format preserves the accuracy of the original data as much as possible. Since it records the topology and the corresponding 3D coordinates from the original models, it completely preserves the information and only has to store the floating data at regular points. We also gain other blank points in the plane from the original triangle patch by shape deformation. Our method ensures a smooth image and a 3D model with no change in the grid shape. The experiments verify the accuracy of the proposed method, and although there are some limitations, the method has a high-quality performance on a common model.

Since the objective of this work is to obtain the vertices of an arbitrary surface map to the integer point coordinates in a grid, we briefly review the related work on geometry images, cutting a surface to a topological disk and obtaining the vertex distribution with blue noise spectral characteristics.

Multichart geometry images (MCGIMs) construct an atlas to map the surface piecewise onto charts of an arbitrary shape^4^. PolyCube-Maps is a seamless texture mapping method that creates a correspondence between 3D grids and multiple cubes to naturally generate geometry images^5^. Adaptive geometry images (AGIMs) use a multiresolution grid to achieve peak signal noise ratio (PSNR) gain^6^. Spherical parameterisation is a robust technique for parameterising a genus-0 surface onto a spherical domain^7^. Gauthier’s method is applicable to arbitrary meshes, but it converts them to genus-0 meshes^8^. Feature-preserving triangular geometry images detect feature curves to constrain vertex sampling^3^. Differential geometry images extend the differential coordinates to image space, which can preserve local details more precisely, though high genera are not mentioned^9^. Connectivity-preserving geometry images (CGIMs) map a triangular mesh onto a rectangular regular array of an image whose reconstructed mesh produces no sampling errors except for round-off errors over the coordinates of vertices^10^. Sinha et al. converted the 3D shape into a authalic parametrization geometry image (APGIM) so that standard CNNs can directly be used to learn 3D shapes^11^. Most geometry images use PSNR to assess the correspondence between the plane and the 3D model^12^.The CGIMs results in the minimum reconstruction error because it intrinsically preserves the connectivity of the original mesh by allowing each vertex to appear repeatedly in an image array. However, resampling or repeating the vertex information on regular points changes the topological correspondence relationship between the 2D planar mesh and the 3D surface.

Erickson and Har-Peled proposed a theoretical method for cutting a mesh of an arbitrary genus into a disk^13^. They defined a cut graph whose removal transformed the surface into a topological disk and constructed a cut graph for an arbitrary polyhedral surface using a breadth-first search of the dual graph^14^. Gu et al. described a method for automatically determining a cut for a 2-manifold triangle mesh^1^. Sheffer et al. found the shortest loop connecting a mesh vertex to itself using front propagation^15^. Ben-Chen et al. obtained the disk topology by determining the 3D curvature of a small number of mesh vertices^16^. Patanè et al. established smooth cut-graphs by cutting the topological handles along the meridian loops of the saddle points of a scalar field^17^. Dey et al. calculated the optimal handle and tunnel loops using a Reeb graph. Poranne et al. interactively cut the mesh parts and join seams^18,19^. Poranne interactively cut the mesh parts and join seams^20^. Chai et al. cut the mesh along the handles and filled the holes in high genus models^21^. Canpen et al. cut the surface into a set of topological disks using a specific cut graph that only had 3- and 3-degree nodes^22^. Although obtaining nontrivial loops was not optimal in Gu’s method, it was efficient. The objective of our proposed method is to automatically obtain the parameterisation boundary. This boundary is mapped using the cut-node method, and the edge of the pairs is recorded to ensure that no cracks occur on the surface. We propose a simple method based on Gu’s algorithm for obtaining the cut graph. We automatically cut the 3D mesh along the constructed Euler circuit to transform the surface into a topological disk.

Floater and Hormann perform a one-to-one mapping from a parameter domain to the surface^23^. The vertex in a planar triangulation to be expressed as a convex combination of its neighbouring vertices was obtained using the mean value theorem for harmonic functions simplified for parameterisation^24^. After the Floater parameterisation, the surface maps to a rectangular grid in the parameter plane. However, in conformal shape representation, it is almost impossible to preserve the topological relationship of the original triangles while keeping the vertices at the integer coordinates of the regular grid. Blue noise sampling can be used to obtain the blue noise characteristics of the vertex distribution by placing the points at suitable locations within the polygon boundary^25^. Blue noise resampling substantially improves the fidelity to the initial surface, but it changes the topology of the original mesh. Capacity-constrained delaunay triangulation (CCDT) does not change the topology of the triangles before the second triangulation step^26^. We use the geometric optimisation of CCDT to obtain nearly evenly distributed vertices and move vertices to the integer coordinate position. Subsequently, we use a grid relaxation algorithm to reduce the image size and minimise the occurrence of degenerate triangles in the previous operation.

In this study, we propose a novel topology-preserving 2D representation of not only genus-0 3D surfaces, but also of a high genera. The goal is to reconstruct a complex closed surface enclosing a 3D object. The three channel pixel values of the 2D mesh store xyz floating point coordinates, and no overlap vertex or intersection triangular faces appear. Thus, all of the geometric information of the 3D mesh is retained completely. The image-stored 16-bit data format per channel can also rebuild the original 3D structure with a high PNSR. Moreover, the shape-preserving filling of image blank points allows the 2D image processing algorithm to try to introduce 3D mesh processing, such as 3D mesh segmentation. Since TPGIM is a 2D regular matrix and the pixel values are integers, is the process of finding the points in the image and stitching the cut boundary is very fast. Our method makes the irregular 3D mesh almost completely represented by 2D regular images.

## Result

### High PNSR geometric reconstruction by the TPGIM

Our method uses 2D arrays to store 3D geometry as much as possible. There are three main advantages of TPGIM. One is that 2D arrays can be easily stored in the GPU memory and preloaded as textures for real-time rendering. Second, TPGIM has higher geometric reconstruction accuracy because the vertices on the 2D grid are moved from the original vertices, rather than the sampled vertices. Third, the 2D processing algorithm can be easily applied to process the 3D mesh.

**Figure 1:**
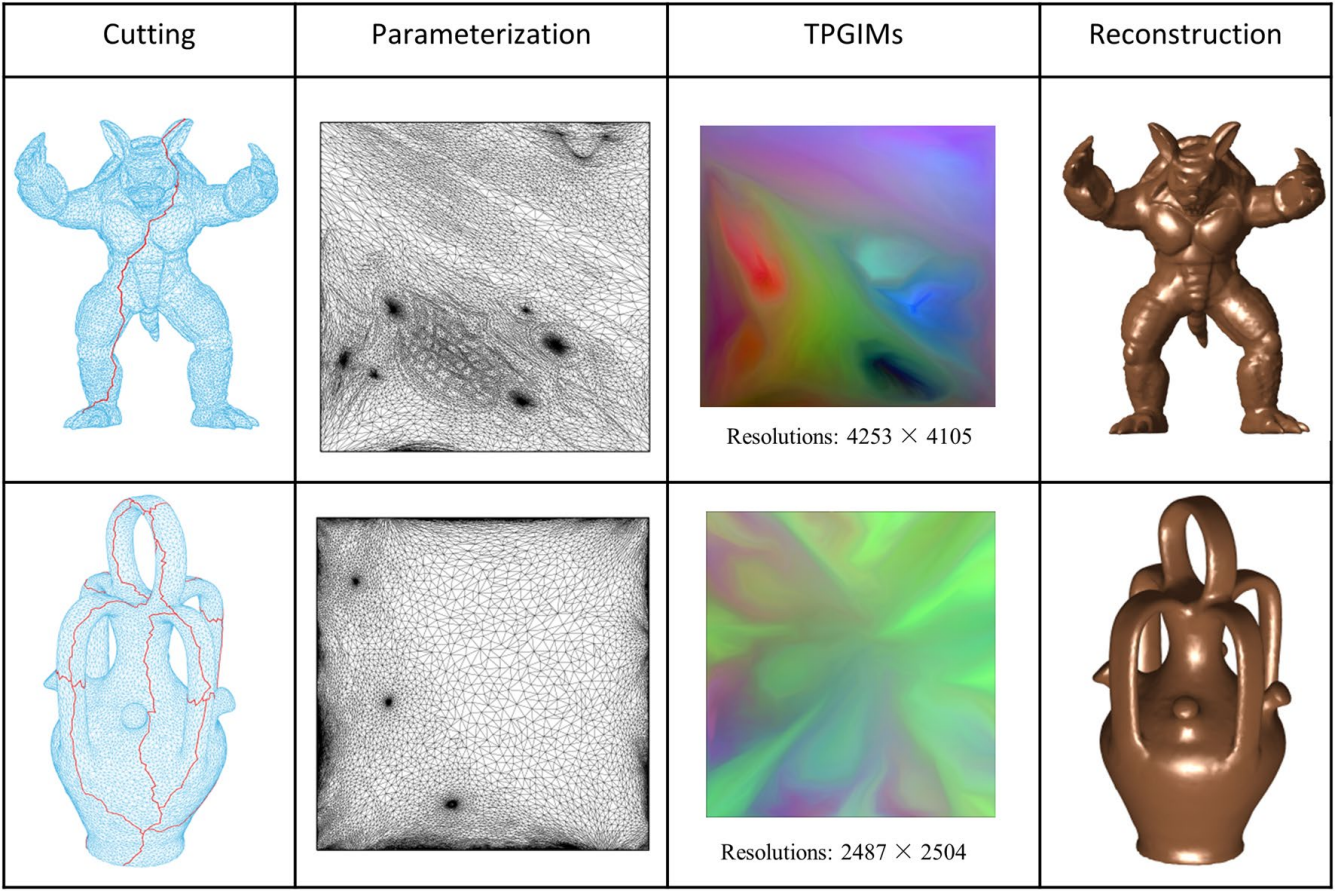
3D models and its TPGIMs.

Figure 1 shows the results of several key steps of our method on two public 3D meshes. Specifically, the first column marks the cutting path on the original mesh, the second column is the set of parameter points of the 3D mesh by mean value coordinates, the third column contains their TPGIM, and the fourth column contains the surface reconstruction from the TPGIM.

We used the peak signal-to-noise ratio (PSNR) to determine the reconstruction accuracy of several geometric images. The PSNR is defined as

1 $$\begin{aligned} PSNR=20log_{10}(peak/d) \end{aligned}$$

where the peak is the bounding box diagonal and d is the symmetric Hausdorff root mean square (RMS) error between the original mesh and the reconstructed geometry image. There is little difference between the reconstructed and the original models, and meshes with high genera are also applicable.

The PSNRs obtained from different algorithms described in the literature are listed in Table 1. The horizontal bar indicates that the original paper was not available. Few algorithms provide experimental results of high genus meshes. We obtained a higher PSNR than the other methods, whether genus-0 or a high genus mesh. The gain of PSNR is increased by 10 db in the genus-0 meshes on average and 42 db in the children of the high genus meshes compared with^8^. Remarkably, we efficiently reduced the reconstruction error and therefore obtained a higher PSNR than the others.

**Table 1:** Reconstruction error (PSNR) comparison.

Examples	Genus number	PNSR of^1^	PNSR of^7^	PNSR of^6^	PNSR of^8^	PNSR of us
bunny	0	78.2	79.8	86.7	–	103.1
venus	0	80.7	83.4	86.7	–	92.83
armadillo	0	72.5	72.0	–	–	107.6
cow	0	78.0	75.5	–	–	99.1
holes3	3	75.2	–	–	–	105.2
children	8	–	–	–	46.08	88.5

More comparison is shown in Table 2. We executed our TPGIM and APGIM on 9 meshes in the same environment. We implemented the complete algorithm in MATLAB 2018. Our experiments were performed on a desktop PC with a 2.30 GHz Intel Core i5-8300 and 8 GB of RAM. Table 2 includes the number of genera, number of faces, a comparison of the computational efficiency of our method and the method in APGIM, as well as the reconstruction time from TPGIM and import time from the 3D model directly.

**Table 2:** Computational efficiency comparison.

Models	Genus	Tris	APGIMs^11^		TPGIMs		Import time(s)
			Generation(s)	Reconst(s)	Generation(s)	Reconst(s)	
bunny	0	22490	166	0.13	812	4.14	8.81
teddy	0	21500	75	0.14	966	2.45	5.01
venus	0	100000	176	0.18	4089	25.7	73.1
Armadillo	0	30000	119	0.15	1340	5.95	14.91
Cow	0	23216	49	0.14	1362	4.05	5.35
Holes3	3	4000	201	0.16	77	0.58	1.86
Fertiliy	4	24972	416	0.2	2146	3.04	5.88
Botijo	5	29734	402	0.16	1308	4.15	7.14
Children	8	23972	266	0.16	1946	4.52	5.64

Table 2 shows that TPGIM had a much longer running time than APGIM, especially for genus-0 models with large sizes. TPGIM also had a longer running time in the high genus model but a small gap compared with the genus-0 models. This is because TPGIM spends much time avoiding violating the constraint of topology preservation. We admit the lower efficiency of TPGIM, as such high-resolution results of TPGIM are unavoidable. However, we have obvious advantages in reconstruction accuracy, which means that the original 3D can be fully represented by TPGIM.

As seen from the reconstruction time column in Table 2, the greater the number of vertices, the greater the time gap between reconstructing the 3D mesh from TPGIM and importing the 3D mesh directly. The 100000 faces venus only takes 25.7 seconds, while it takes 73.1 seconds from 3D mesh importing with Obj format. This is because our vertex index is calculated directly using a 2D regular structure, and removing duplicate points to sew the model on 3D integer coordinates occurs quickly. Since the image can almost completely represent the mesh, it is worthwhile to spend some time offline to obtain the TPGIM.

### 2D image processing algorithm applied to the 3D model

TPGIM is suitable for attempting to apply an image processing algorithm that does not change the image size to process 3D models (wavelet-based encoder is not suitable). The structural storage of images has considerable advantages and can use a mature 2D image processing algorithm to solve complex 3D problems. To demonstrate the research potential of TPGIM, we use a 2D image processing algorithm for 3D mesh segmentation.

**Figure 2:**
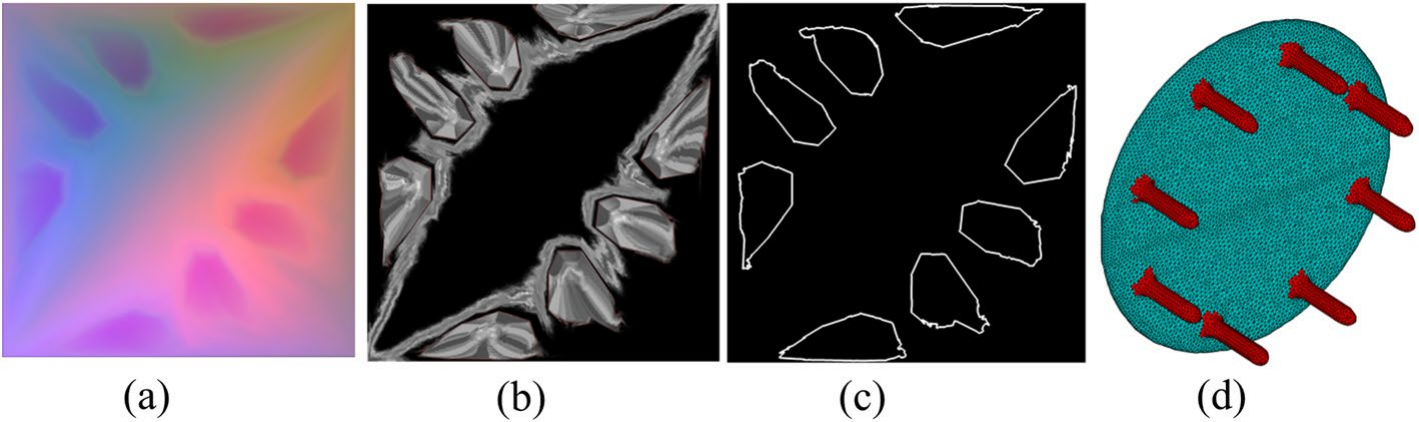
3D mesh segmentation using TPGIM.

As shown in Fig. 2, the results of its 2D image segmentation can be applied to the 3D model. Figure 2a shows the TPGIM of a table. It has eight obvious enclosed areas, which are exactly the eight legs of the table. Although we can see the boundaries of these eight regions with the naked eye, the results obtained by the image edge detection algorithm are not ideal. However, TPGIM stores not only 3D coordinates, but it also stores other attributes of the vertices. A verifies that the curve along the minimum value of the negative principal curvature of the mesh is the best position for 3D mesh segmentation.

Figure 2b shows the result of calculating the vertex curvature by^27^ stored on the image. The minimum value of the principal curvature of the vertex is taken as the absolute value^28^, interpolated and stored on the regular TPGIM. The higher gray value area in the middle of the grey image is the desktop boundary of the round table, and the 8 table legs. The boundaries of the eight leg areas are very clear.

Next, we use the region-based active contour model with 500 iterations^29^, which is a classical 2D image segmentation algorithm to obtain closed segmentation boundaries. The selection of the target contour is based on setting the threshold of the number of vertices contained in the contour. Figure 2c shows the selected 8 contours, which have been as close to the boundary of the target area as possible. After the vertices surrounded by 8 closed contours are rebuilt into the 3D model, part of the green patch is shown in Fig. 2d. The eight legs of the table are divided, and the accuracy of segmentation on the 2D image affects the segmentation of the 3D model. This example shows the potential of our TPGIM to process 3D models.

## Discussion

The results presented here provide a step forward by demonstrating the accurate 2D regular structure parameterisations of 3D mesh by preserving topology. The results also provide insights into how well a TPGIM can perform as a replacement of a 3D mesh. We found that we could create an efficient TPGIM on a wide variety of models. However, models of much higher genera or with long and narrow paths should need large image sizes to ensure no change in topological structure. For these models, the cutting path can only be adjusted manually to obtain an acceptable image.

To study the applicability of this algorithm, we use unified parameters to generate TPGIM on the dataset of 380 models of Meshsegbenchmark. Several failure examples help us analyse the applicability of this method. We define an image width greater than or equal to 8000 as a failed image. A total of 280 models are successful directly, 55 models are generated automatically after simplification, and 45 models need to manually adjust the cutting path. The manual adjustment models have high genus or long and narrow shape components (such as the elongated leg of the spider). Therefore, we suggest that the model shape is simple or within approximately 20000 vertices, which can be used automatically with the proposed algorithm. Otherwise, it is preferable to perform appearance-preserving simplification first or manually adjust the cutting path to obtain the right result.

Important aspects of the approach to accurately represent a surface using an image make the study different from previous studies. First, we demonstrated the process of obtaining a smoother path to cut a 3D mesh into a topological disk. This approach can be used as the first step for surface parameterisation. Second, after parameterising the 3D mesh to the plane, the CCDT without retriangulation is adopted to obtain a vertex distribution as uniform as possible to realise that the vertices can move to integer points while maintaining the topology. Third, the grid relaxation algorithm moves the vertices to integer point coordinates. The method is efficient for establishing a correspondence between the 3D surface and the 2D plane. Our methodology is robust for all of the surfaces we tried it on. However, TPGIM also shows the potential of using a 2D image processing algorithm to process 3D models because the image can store not only 3D coordinates, but also any of the other vertices’ attributes, such as vertex curvature, shape index, and normalcy.

Future research on TPGIM must address technical challenges, such as to automatically obtain suitable cutting for reducing the size of the image and the best method to handle meshes with a high genus or long and narrow shape components. Future research should also continue to seek insights into 3D model processing or analysis using 2D image processing algorithms.

## Methods

This paper proposes a topology-preserving 2D representation of a 3D surface mesh. This method can store the surface mesh structure in the geometric image and is suitable for high genus mesh. The mesh stored in this way can also be reconstructed into the original 3D structure with a high PNSR.

We provide a theoretical background by introducing cutting a surface into a topological disk and describe our Euler-circuit cutting method. Then, the surface is mapped to a square in the 2D domain from 3D meshes by the parameterisation method of the mean value coordinates. To obtain nearly uniform points by the first step of the CCDT, we first optimise the parametric vertices’ geometry positions. Then, a relaxed grid is constructed to move all vertices to integer positions similar to topology preservation without folding. The points in the blank position of TPGIM are filled in the transformed data between the deformed plane triangle and the rotated original 3D triangle.

### Euler circuit cutting to obtain a topological disk

Gu’s method is used to obtain the cutting path of the 3D mesh. While a mesh of genus-0 is closed, the boundary of the parameterisation is two adjacent mesh edges. For the high genus meshes, Gu applied two phases to find cut after removing a single seed triangle from the mesh. In the first phase, a topological disk that includes all of the faces of the mesh is removed. In a second phase, the edge trees are trimmed away, leaving just the connected loops. Finally, the serrated cut is straightened by computing a constrained shortest path that connects its two adjacent cut-nodes.

**Figure 3:**
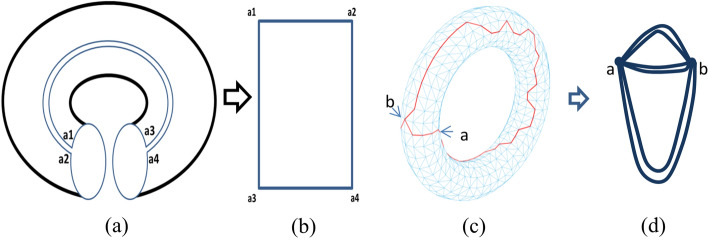
Unwrap the torus into a plane and construct an undirected graph from a cut path.

#### Initial cut

We chose a long cut as the parameterisation boundary of genus-0 meshes and used a cutting path based on Gu’s method as the initial cut for high genus meshes. The cut $\rho$ was calculated using the MATLAB source code based on Gu’s C++ code of a cut graph developed by Wen^30^. Since the obtained cuts $\rho$ are not in consecutive order, the serrated cuts $\rho$ cannot be straightened. The triangles on the boundary of the rectangular parameterisation change into a straight line if the mesh is cut along the edges. It is also easy to create errors when the connection degree of the cut graph is greater than 2. Thus, we later use an Euler circuit cutting algorithm to change the surface to a simply- connected mesh with a single boundary $\rho$′ after obtaining the cut $\rho$.

Our method does not deal with the boundary and the interior separately because the boundary between each cut-path and its mate is the parameterisation boundary. As long as we construct this boundary in the 3D mesh, it is easy to map between the cut $\rho$′ and the boundary of the unit square *D* using floater parameterisation. This process is illustrated in Fig .3a,b, the connections between a1 and a2, a3 and a4, a1 and a3, and a2 and a4 are cut-path pairs. After unwrapping the torus into a plane along the boundary, all spatial points are mapped inside the boundary, regardless of whether the boundary $\rho$′ is optimal. Note that the vertices will degenerate into the boundary line on the plane when the edges of the triangle are located on the boundary. Since degenerate triangles may also occur in subsequent operations, we will solve them in a unified manner later.

#### Euler circuit cutting

We simplify the cutting path ρ as an undirected graph to obtain the boundary $\rho$′ automatically. As shown in the Fig. 3c, $\rho$ has 3-degree nodes {*a*,*b*}. We use all points set *v* with the degree greater than 2 as the vertices and the double path between the points as the edges to form an undirected graph *g* (as shown in the Fig .3d) while the path $\rho$ and its mate to form the boundary $\rho$′. An Euler circuit must exist in the graph *g*. The Euler circuit establishes a sequence so that the cutting boundary can be accessed sequentially, so as to reduce the sawtooth of the cutting path $\rho$′. After obtaining the Euler circuit, we calculate the shortest path between the consecutive 3 points on the cutting path $\rho$′ following the sequence by Euler circuit and iterate this process several times. Note that the paired paths should be consistent when updating the boundary. However, this operation is not performed at the adjacency vertices of points set *v* to prevent misalignment. Then, we perform patch separation of the mesh along $\rho$′, making the slicing surface simply connected with a single boundary.

**Figure 4:**
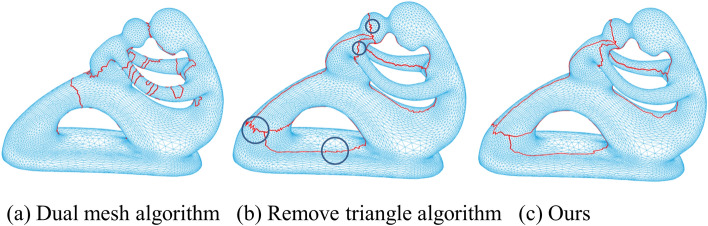
Three different cutting paths.

Wen developed 2 algorithms for obtaining a cut graph. The first is the implementation of the algorithm in the book^31^, and the other is the translation of David Gu’s C++ code^30^, which is much faster than version 1. We conducted experiments on Fertility. As shown in Fig. 4a, there are too many circular paths to use the dual mesh algorithm. Version 2 resulted in many zigzag shapes (the boundary point in the blue circle in Fig. 4b. The proposed Euler-circuit cutting method provided the best results for determining the cut-path of the TPGIM (Fig. 4c)).

Finally, we delete the redundant vertices with no connection information and perform Floater parameterisation of this mesh with a closed boundary. We use the mean value coordinates algorithm to map the surface to the unit rectangle using 3D coordinates. Each point, except the boundary, is a convex combination of adjacent points. We solve the sparse linear equation to map the points in the 3D mesh to the plane square after specifying the rectangular boundary coordinates.

### Moving vertices to the integer position

We need to move the vertices to the integer coordinate positions while preserving the topology and obtaining a small grid of the TPGIM. The vertex distribution determines whether a vertex can be successfully moved. The vertex distribution with blue noise spectral characteristics is very useful for moving the vertex to integer coordinates. The purpose is to obtain a grid as small as possible while preserving the topology.

#### Gaining nearly uniform distribution

CCDT is proposed to generate a nearly uniform point distribution. The first phase optimizes the geometry and does not change the topological structure. Denoting $a_i=(y_i-y_{i+1})$,$b_i=(x_{i+1}-x_i)$and $c_i=(x_iy_{i+1}-x_{i+1}y_i)$, we get $A_i=a_ix+b_iy+c_i$. $(x_{i+1},y_{i+1})$, $(x_i,y_i)$and (*x*, *y*) are vertices of triangle.*A* is the area. Xu used the equation for the area of a triangle and optimized the vertex position by minimizing the variance

2 $$\begin{aligned} \sum _{i=1}^k\left( A_i-\frac{1}{k}\sum _{j=1}^k(A_j)\right) ^2 \end{aligned}$$

The quadratic equation provides the *x* and *y* coordinates in a $2 \times 2$ linear system, *k* is the number of triangles.

Since we do not perform the second step (connectivity optimization), we obtain a nearly uniform distribution. If the point distribution is highly uniform, large obtuse triangles will occur. In contrast, if the point distribution is not sufficiently uniform, the image size will be very large. If a triangle with an excessively large angle is generated when moving a vertex, control parameters can be added to the geometry optimization to stop the movement of this point. However, this will increase the geometry image size. This approach is used when the vertices cannot be moved to an integer position after multiple iterations.When the grid is large, too much stretching occurs when all points are moved to an integer position. We use an energy threshold of 1*e*-3 in ordinary circumstances.

#### Relaxed grid

After obtaining a near-uniform point distribution, we construct a grid for the moving vertices to the integer coordinates. The neighborhood distance is defined as:

3 $$\begin{aligned} S=\min \limits _{A}\{\max \limits _{B}\{neighbor_x,neighbor_y\}\} \end{aligned}$$

where B is the horizontal and vertical distance from the adjacent point, and A is the set of B obtained from adjacent points. We set the grid interval to the minimum S for all vertices and divide the coordinates of each vertex by the interval. This approach ensures at least one interval in the X or Y direction between each point and the adjacent points.

We moved each point to the nearest integer coordinate. It is crucial in subsequent operations that the final result cannot be repeated and folded. No folding occurs if the stretching and compression are uniform when we pull the boundary to an integer position. We developed a criterion to prevent folding when moving the internal points. We calculated the area of all triangles after the vertex movements. If the area is greater than the rectangular area, it means that a fold has occurred and the vertex cannot be moved.

4 $$\begin{aligned}&\sum _{i=1}^k\lambda _iv_i=v_0 \end{aligned}$$

5 $$\begin{aligned}&\lambda _i=\frac{\omega _i}{\sum _{j=1}^k\omega _j}, \omega _i=\frac{tan(\alpha _{i-1}/2)+tan(\alpha _i/2)}{\Vert v_i-v_0\Vert } \end{aligned}$$

Equations (4, 5) ensures that the xy positions do not repeat after rounding the numbers. The average coordinates of the internal points are calculated. If there are integer points around the calculated mean value coordinates, it is be moved directly. We find an integer point and adjust the CCDT using multiple iterations. There is no priority of the points in the next iteration. If a vertex cannot be moved to an integer point, it is assigned to a neighbouring point.

At this time, it is not guaranteed that all points can be moved to the integer position. We do not exclude moving repeatedly or aligning 3 points to form a straight line. Therefore, we stretch the vertex coordinates three times to make room for adjusting the vertex position, which ensures 8 vacancies around each point. After correcting the vertex position, we deleted the blank line and emptied the columns to prevent folding. We call this the relaxation method, i.e., we perform iterative stretching and compression until points cannot be moved anymore, and there are no degraded triangles. This approach continuously increases the mesh size by adjusting the vertices and shrinking the mesh to remove blank spaces until convergence is reached. It should pay attention to the settings to stop loss in practice because there is no guarantee that it always converges.

If there are duplicate points or degenerate triangles, all points are moved to integer points. The recovery of the repeating points and degraded triangles is performed separately. All 8 slots are optional when points are moved repeatedly. When the triangle changes to a straight line, we calculate the midpoint of the line as a moving point. We determine the movement direction by finding an undegraded triangle in the neighbourhood points. There are four potential cases: horizontal line, vertical line, slope > 0, and slope < 0. In each case, there are 2 directions and 3 slots. In case one, the vertex can be moved to the upper left, top, upper right 3 points, and bottom 3 points. In case two, the vertex can be moved to the upper left, left, lower left 3 points, and right 3 points. In case three, the vertex can be moved to the upper left, left, top 3 points, and opposite 3 points. In case four, the vertex can be moved to the upper right, right, top 3 points, and opposite 3 points.

**Figure 5:**
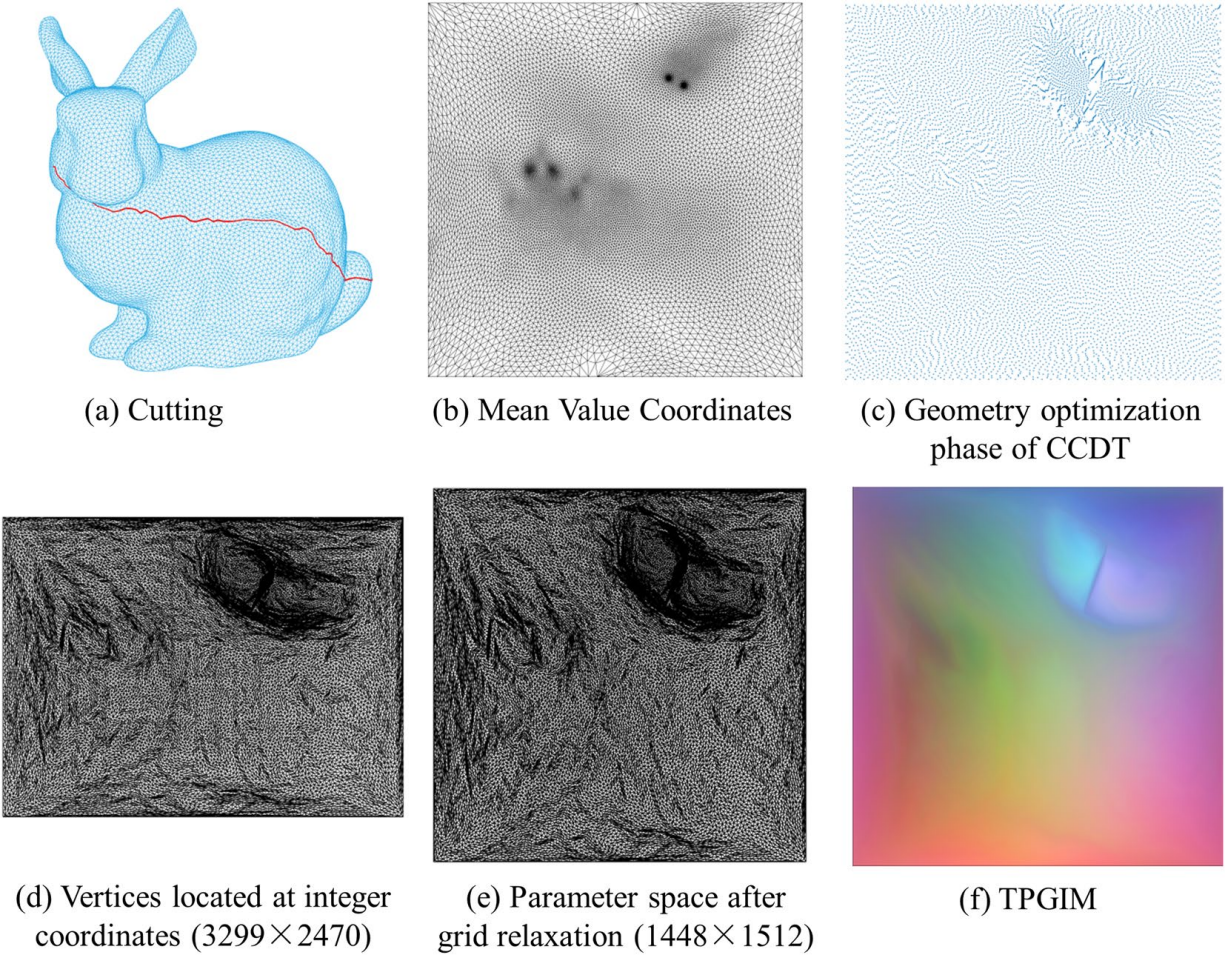
The whole pipeline of moving vertices to the integer position.

After that, most of the blank rows and columns are redundant. We alternately delete the random rows and columns to prevent creating a narrow rectangle. The compressed operation ensures that redundant rows and columns are eliminated under the constraint of ensuring no new degradation.

Figure 5 is an explanation. First, a cutting path of the bunny is obtained. The path of the genus-0 model can freely select a long cutting line. Generally, the path between the two points with the farthest coordinates in the model is selected by default. Second, the mean value parameterisation is used to gain one-to-one mapping between the 3D mesh and unit rectangle. Third, the CCDT without retriangulation is used to obtain a nearly uniform point distribution as shown in Fig. 5c. Fourth, all vertices are moved to integer points while maintaining their topological relationship. If duplicate points or degenerate triangles exist, the vertex coordinates are stretched three times to make room for adjusting the vertex position. Rows and columns are randomly added for verifying the relaxation algorithm in Fig. 5d. Fifth, a compression operation is performed to reduce image redundancy. As shown in Fig. 5d,e, 3299 times 2470 of the image is changed to 1448 times 1512. The fourth and fifth steps may iterate several times when repeated points and degenerate triangles appear. Finally, TPGIM is generated by shape deformation, which is described in the next section.

### TPGIM generation by shape deformation

Geometric images need to store 3D vertex coordinates in the three channels of the RGB image. The original 3D vertices are all integer values in the final images, and the topological relationships are recorded independently. We use the shape deformation of each original triangle to realise that the reconstructed 3D mesh does not change the shape of the original model. In this way, the blank position in the image is smooth and consistent with the original surface. For vertex curvature, vertex normal and other features, a general colour interpolation algorithm is used to fill the blank position in the image.

To fill the blank position by shape deformation, the integer vertex coordinates are calculated in each patch on the planar mesh. Next, we rotate the triangle to the plane by calculating the rotation matrix of the patch normal and the z-axis of the original mesh. The patch in 3D and the plane patch are two triangles with different shapes. We then calculate the affine matrix by shape deformation to transform the data between the plane triangle and the rotated original triangle. Finally, the plane points are converted back to 3D coordinates using the rotation matrix.

An image with floating-point data can store the information of the original model. However, the number of patches increases sharply as the image size increases. If we calculate the coordinates of the blank points without triangulating each patch, the plane image of the discrete points is consistent with the original model, and the operation is performed efficiently. Therefore, triangulation is only performed to show that the shape of the final model is the same as that of the original model. We only need the image and topological relationship for mesh reconstruction. Since the TPGIM stores data in a 2D array, the coordinates of the image can be calculated according to the serial number, and the original mesh can be completely restored as long as duplicate points are removed and the topological relationship is updated.
